# Dynamic Modeling of *Carnobacterium maltaromaticum* CNCM I-3298 Growth and Metabolite Production and Model-Based Process Optimization

**DOI:** 10.3390/foods10081922

**Published:** 2021-08-19

**Authors:** Cristian Puentes, Amélie Girardeau, Stephanie Passot, Fernanda Fonseca, Ioan-Cristian Trelea

**Affiliations:** 1 INRAE, AgroParisTech, UMR SayFood, Université Paris-Saclay, F-78850 Thiverval-Grignon, France; cristian.puentes@centralesupelec.fr (C.P.); amelie.girardeau@inrae.fr (A.G.); stephanie.passot@inrae.fr (S.P.); fernanda.fonseca@inrae.fr (F.F.); 2 CentraleSupélec, LGPM, Université Paris-Saclay, F-91192 Gif-sur-Yvette, France

**Keywords:** *Carnobacterium maltaromaticum*, modeling, microbial growth, optimization, fermentation

## Abstract

*Carnobacterium maltaromaticum* is a species of lactic acid bacteria found in dairy, meat, and fish, with technological properties useful in food biopreservation and flavor development. In more recent years, it has also proven to be a key element of biological time–temperature integrators for tracking temperature variations experienced by perishable foods along the cold-chain. A dynamic model for the growth of *C. maltaromaticum* CNCM I-3298 and production of four metabolites (formic acid, acetic acid, lactic acid, and ethanol) from trehalose in batch culture was developed using the reaction scheme formalism. The dependence of the specific growth and production rates as well as the product inhibition parameters on the operating conditions were described by the response surface method. The parameters of the model were calibrated from eight experiments, covering a broad spectrum of culture conditions (temperatures between 20 and 37 °C; pH between 6.0 and 9.5). The model was validated against another set of eight independent experiments performed under different conditions selected in the same range. The model correctly predicted the growth kinetics of *C. maltaromaticum* CNCM I-3298 as well as the dynamics of the carbon source conversion, with a mean relative error of 10% for biomass and 14% for trehalose and the metabolites. The paper illustrates that the proposed model is a valuable tool for optimizing the culture of *C. maltaromaticum* CNCM I-3298 by determining operating conditions that favor the production of biomass or selected metabolites. Model-based optimization may thus reduce the number of experiments and substantially speed up the process development, with potential applications in food technology for producing starters and improving the yield and productivity of the fermentation of sugars into metabolites of industrial interest.

## 1. Introduction

*Carnobacterium maltaromaticum* is a psychotropic species of lactic acid bacteria widely found in food such as dairy products, fish, and meat. It is a Gram-positive, facultative anaerobic bacterium, able to grow at alkaline pH (up to 9.6) [1,2].

In the food industry, *C. maltaromaticum* has potential applications related to health protection and organoleptic properties. These include the biopreservation of food, by inhibiting the growth of foodborne pathogens such as *Listeria* sp. in cold conditions, and the development of flavor in ripened cheese varieties [2,3,4].

This lactic acid bacterium may also be used as a biological indicator in time–temperature integrators (TTI): ‘smart-labels’ that monitor the time–temperature history of chilled products throughout the cold-chain [5,6]. Concentrates of the strain CNCM I-3298 have been selected as inoculum for TopCryo^®^ labels, the only biological TTI that has been taken to market to date. A pH decline of the label medium, associated with bacterial growth and acid production, produces an irreversible color change from green to red as an indication to the consumer about the spoilage of the food to which the TTI is attached [7].

In these applications, *C. maltaromaticum* concentrates produced by fermentation may be used alone or in association with other microorganisms. Some experimental studies on *C. maltaromaticum* fermentation under different culture conditions have been reported in the literature [3,4,6,7,8,9,10,11,12,13,14]. The effect of temperature and pH on the acidifying activity was evaluated and modelled by Girardeau et al. [7]. However, there is a lack of knowledge on the characterization and optimization of Carnobacteria growth and production of various metabolites such as acids or flavor compounds in a bioreactor.

**Figure 1:**
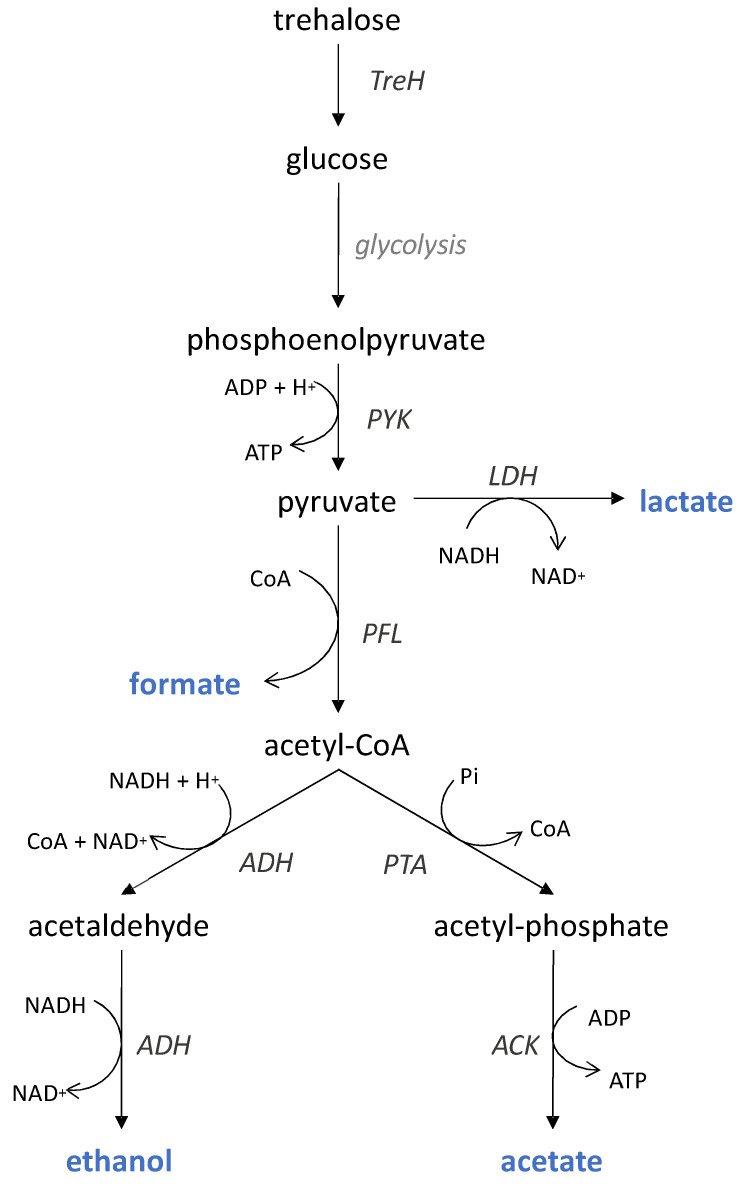
Mixed-acid fermentation pathway likely used by *C. maltaromaticum* to ferment trehalose. End products are shown in blue. ACK, acetate kinase; ADH, acetaldehyde dehydrogenase; LDH, lactate dehydrogenase; PFL, pyruvate formate lyase; PTA, phosphate acetyltransferase; PYK, pyruvate kinase; TreH, neutral trehalose. Adapted from [19,20,21,22,23].

*Carnobacteria* are considered to be homofermentative lactic acid bacteria that produce lactic acid from glucose, with pyruvate as a central metabolic intermediate (via the Embden–Meyerhof pathway) [15,16,17]. However, pyruvate may be alternatively converted to acetate, ethanol, formate, and CO_2_ [16,18] under anaerobic conditions and substrate limitation [19], arising for example at the end of fermentation [20]. The production of organic acids by Carnobacteria is also strain-dependent [8,16,21]. A recent study reported that lactic, formic, and acetic acids are key organic acids produced by *C. maltaromaticum* in a meat juice medium [22], indicating that this microorganism has the enzymatic machinery to perform mixed-acid fermentation (Figure 1).

For optimization purposes, modeling has proven to be a powerful tool, enabling the exploration of a wider range of operating conditions while minimizing cost, compared with the experimental approach [24,25,26,27,28,29]. To our knowledge, the only dynamic model dealing with *C. maltaromaticum* strains has been published by Ellouze et al. [6]. That research was oriented towards a biological TTI setting associated with a sausage-like packaging instead of a bioreactor and taking into account lactic acid as the single metabolite.

The aim of this study was thus to develop and validate a dynamic model predicting the impact of fermentation conditions (temperature and pH) on the growth and bioconversion fermentation dynamics of *C. maltaromaticum* CNCM I-3298 using trehalose as a carbon source and considering the four main identified metabolites: formic acid, acetic acid, lactic acid, and ethanol. This study was conducted as part of a research project on the production and conservation *of C. maltaromaticum* concentrates. In that context, the growth of *C. maltaromaticum* was tested in different sugars: glucose, maltose, mannitol, and trehalose, with similar growth rates. Trehalose was chosen in this study because this molecule is known for its ability to protect cells during bacterial stabilization processes (freeze-drying in particular). Therefore, the residual trehalose (not consumed during fermentation) could be used as cryoprotectant after production of bacterial concentrates.

The model development involved four major steps, presented in Section 3: derivation of the main governing equations based on the known mixed-acid fermentation pathway, mass balances, and kinetic rate expressions (Section 3.1); parameter identification for each fermentation experiment (Section 3.2); construction of response surfaces of the calibrated parameters as a function of temperature and pH (Section 3.3); and final validation of the complete model. The resulting model is shown to be a useful tool in determining the optimal conditions for producing bacterium concentrates in bioreactors and for assessing the productivity of the bioconversion fermentation of sugars into metabolites of potential industrial interest (Section 4.4).

## 2. Materials and Methods

**Figure 2:**
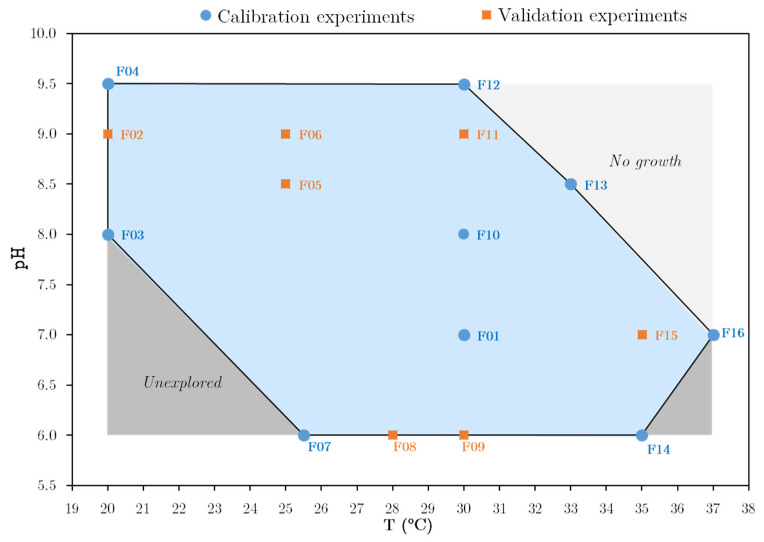
Operating conditions of *C. maltaromaticum* CNCM I-3298 fermentation experiments.

Data used to calibrate and validate the model were partially reported in a previous study, in which a modified central composite experimental design was carried out to study the effect of operating conditions on the technological properties of *C. maltaromaticum* CNCM I-3298 [7]. Sixteen lab-scale fermentations (hereafter named F01 to F16) were performed using a wide range of regulated operating conditions (Figure 2): temperature between 20 and 37 °C and pH between 6.0 and 9.5.

Fermentation durations varied between 20 h and 45 h, and the initial conditions were: for biomass (X_0_) 0.077 mol_C_·L^−1^, trehalose (S_0_) between 0.091 mol·L^−1^ and 0.107 mol·L^−1^, and medium volume (V_0_) 3.5 L.

The main fermentation settings and the kinetic measurements are reported below.

### 2.1. Fermentation

#### 2.1.1. Culture Medium and Bacterial Strain

The fermentation medium was composed of the following ingredients for 1 kg of final solution: 40 g of trehalose (Treha™; Tokyo Japan); 10 g of proteose peptone (Oxoid; Waltham, MA, USA); 5 g of yeast extract (Humeau; La-Chapelle-sur-Erdre, France); 5 g of Tween 80 (VWR; Leuven, Belgium); 0.41 g of MgSO_4_ (Merck; Darmstadt, Germany); 0.056 g MnSO_4_ (Merck; Darmstadt, Germany); and water to reach a total of 1 kg of solution. All medium components were sterilized together at 121 °C for 20 min. Fermentations were carried out on *C. maltaromaticum* CNCM I-3298 pre-cultures. Pre-cultures were prepared by inoculating 10 mL of sterilized fermentation medium with 100 µL of *C. maltaromaticum* CNCM I-3298 stock culture and were incubated for 13 to 16 h at 30 °C. An amount of 1 mL of the resulting culture was transferred into 50 mL of fresh medium and then incubated again for 11 h under the same conditions. The resulting culture was then used to inoculate the bioreactor. Inoculation was performed at an initial concentration of approximately 10^7^ CFU mL^−1^.

#### 2.1.2. Bioreactor and Parameter Control

The bioreactor (Minifors, Infors HT, Bottmingen, Switzerland) had a total volume of 5 L and was equipped with a heat mantle and a cryostat for temperature control. It contained 3.5 L of fermentation medium, inoculated with an initial cell concentration of approximately 10^7^ CFU·mL^−1^. Initial pH was adjusted to the desired value with 5 M NaOH or 0.01 M H_2_SO_4_ solutions. During fermentation, pH was controlled to the desired setpoint for each investigated condition (Figure 2) by automatic addition of 5 M NaOH. Culture homogenization was performed with an agitation device set at 150 rpm. Temperature was set according to the investigated operating conditions mentioned above (Figure 2).

### 2.2. Kinetic Measurements

#### 2.2.1. Cell Growth

Cell growth was monitored using an infrared probe (Excell210, CellD, Roquemaure, France) continuously measuring absorbance at 880 nm and storing data every minute. The absorbance data were calibrated in dry weight. Dry cell weight was determined by filtering 10 mL of bacterial suspension (straight out of the bioreactor) through a 0.20 µm polyethersulfone membrane (Supor^®^, PALL Biotech, Saint-Germain-en-Laye, France). The filter was then dried for 24 h at 80 °C. Measurements were obtained in triplicate. Mass concentrations were finally converted to mol_C_ L^−1^ (carbon-mol of biomass per liter), assuming the simplified unit-carbon biomass formula CH_1.8_O_0.5_ [30].

#### 2.2.2. Total Acid Production

Total acid production was determined according to the volume of NaOH solution injected into the bioreactor to maintain a constant pH. The pH was regulated/controlled to set values using the IRIS NT V5 software (Infors, AG, Bottmingen, Switzerland).

#### 2.2.3. Substrate Consumption and Metabolite Production

Trehalose consumption and metabolite production were determined using high-performance liquid chromatography (HPLC, Waters Associates, Millipore; Molsheim, France). HPLC was performed on culture media samples of a few mL, aseptically retrieved from the bioreactor at different times during fermentation and filtered through 0.22 µm pores (Sartorius stedim, Biotech; Göttigen, Germany). Analyses were made using a cation exchange column (Aminex Ion Exclusion HPX-87 300 × 7.8 mm, Bio-Rad, Richmond, VA, USA) at 35 °C. Mobile phase was 0.0005 M H_2_SO_4_, and flow rate was set at 0.6 mL·min^−1^ (LC-6A pump, Shimadzu, Courtaboeuf, France).

HPLC analysis showed that *C. maltaromaticum* CNCM I-3298 produced not only lactic acid but also formic acid, acetic acid, and ethanol in variable proportions according to the fermentation conditions.

## 3. Dynamic Model

The mathematical model was a set of ordinary differential equations implemented in MATLAB R2018b (the MathWorks Inc. Natick, MA, USA). Model parameters and response surface coefficients were identified by nonlinear regression analysis using the Statistic and Machine Learning Toolbox of MATLAB.

### 3.1. Model Formulation

The dynamic model developed in this study combined biochemical knowledge about the metabolism of the selected bacterium and mass balances of the main compounds: substrate, biomass, and identified metabolites. Expressions of specific growth and metabolite production rates included substrate limitation, product inhibition phenomena, and time lags due to microbial metabolism adaptation [31]. The surface response method was used to express the empiric dependence of some model parameters on operating conditions. The model assumed the bioreactor was perfectly stirred and there were no differences between individual cells. It was thus unsegregated and zero-dimensional, predicting average spatial concentrations [32].

Seven state variables were considered: six volume concentrations (biomass [X], trehalose [S], formic acid [F], acetic acid [A], lactic acid [L], and ethanol [E], Figure 1) and the culture medium volume (V). This latter variable varied continuously with the addition of base (NaOH) for pH control but also changed in a discrete way due to periodic sampling for biological and chemical analysis.

Mass balances for the considered metabolites resulted in the following set of differential equations:

(1) $$\frac{d\left[X\right]}{\mathrm{dt}}=\mu_{X}\left[X\right]-\frac{Q}{V}\left[X\right]$$

(2) $$\frac{d\left[F\right]}{\mathrm{dt}}=\pi_{F}\left[X\right]-\frac{Q}{V}\left[F\right]$$

(3) $$\frac{d\left[A\right]}{\mathrm{dt}}=\pi_{A}\left[X\right]-\frac{Q}{V}\left[A\right]$$

(4) $$\frac{d\left[L\right]}{\mathrm{dt}}=\pi_{L}\left[X\right]-\frac{Q}{V}\left[L\right]$$

(5) $$\frac{d\left[E\right]}{\mathrm{dt}}=\pi_{E}\left[X\right]-\frac{Q}{V}\left[E\right]$$

(6) $$\frac{d\left[A_{T}\right]}{\mathrm{dt}}=\frac{d\left[F\right]}{\mathrm{dt}}+\frac{d\left[A\right]}{\mathrm{dt}}+\frac{d\left[L\right]}{\mathrm{dt}}$$

(7) $$\frac{d\left[S\right]}{\mathrm{dt}}=-\left(\frac{\mu_{X}}{Y_{X/S}\;}+\frac{\pi_{F}}{Y_{F/S}\;}+\frac{\pi_{A}}{Y_{A/S}\;}+\frac{\pi_{L}}{Y_{L/S}\;}+\frac{\pi_{E}}{Y_{E/S}\;}\right)\left[X\right]-\frac{Q}{V}\left[S\right]$$

(8) $$\frac{\mathrm{dV}}{\mathrm{dt}}=Q$$

Here, μ_X_ is the specific growth rate (h^−1^); π_F_, π_A_, π_L_, and π_E_ are the specific production rates of four metabolites (h^−1^); and Y_X/S_, Y_F/S_, Y_A/S_, Y_L/S_, and Y_E/S_ are the yield of biomass and metabolites with respect to the substrate (mol.mol^−1^). Q is the experimentally measured rate of NaOH solution (L.h^−1^) added for pH control throughout fermentation.

In Equation (6), [A_T_] is the total acid concentration, defined as the sum of formic, acetic, and lactic acid concentrations. These compounds are assumed to be mainly responsible for the pH change of the liquid medium.

Specific growth and production rates were defined using the Monod law to account for substrate limitation, modified with product inhibition and enzymatic activation factors [33,34,35]:

(9) $$\mu_{X}=\mu_{\max,X}I_{X}E_{A}\frac{\left[S\right]}{K_{\mathrm{SX}}+\left[S\right]}$$

(10) $$\pi_{m}=\pi_{\max,m}I_{m}E_{A}\frac{\left[S\right]}{K_{\mathrm{Sm}}+\left[S\right]}\;\;\;\;m=F,\;A,\;L,\;E$$

In these equations, I_X_ and I_m_ are inhibition factors that depend on the inhibitor concentration. They vary between 1 and 0. Inhibition increases with the inhibitor concentration, and its effect on the specific rate is maximal when the corresponding factor is 0. In this model, progressive inhibition factors of the following form were used [36,37]:

(11) $$I_{X}=\frac{1}{1+{\left(\frac{C_{I}}{K_{\mathrm{IX}}}\right)}^{n}}$$

(12) Im=11+CIKImp    m=F, A, L, E

K_IX_ and K_Im_ represent characteristic concentrations of the inhibitors (mol L^−1^) such that the corresponding rates (μ_X_ and π_m_) are reduced by a factor of 2 compared with the absence of inhibitor, *n* and *p* are shape factors, and C_I_ is the concentration of the inhibitor. Since all the metabolites were produced in similar proportions and no biochemical knowledge about their relative inhibiting nature was available, C_I_ was simply defined as the sum of the four metabolite concentrations:

(13) $$C_{I}=\left[F\right]+\left[A\right]+\left[L\right]+\left[E\right]$$

**Figure 3:**
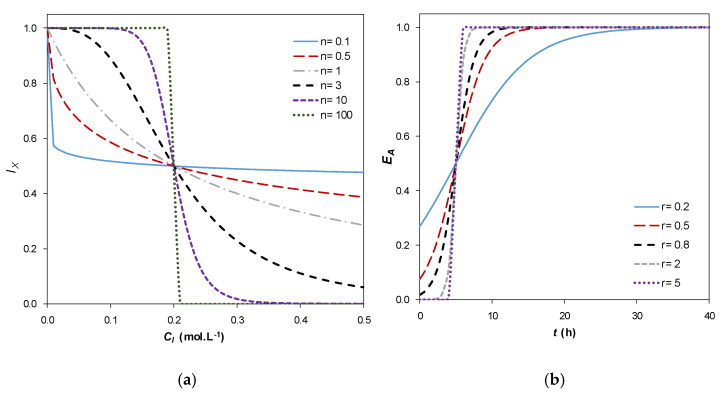
(**a**) Example of inhibition factor I_X_ as a function of C_I_ for different n values and K_IX_ = 0.2 mol L^−1^. (**b**) Example of enzymatic activation factor E_A_ as a function of t for different r values, and t_lag_ = 5 h.

To illustrate the role of the shape factor n, Figure 3a depicts the evolution of I_X_ with C_I_ for different n values and a lag-time of 5 h. A more or less sharp change in the inhibition factor occurs around the characteristic inhibitor concentration, C_I_ = K_IX_. The significance of the shape factor p is similar.

The enzymatic adaptation factor E_A_ is an empirical representation of the lag time, a period of adaptation to the culture environment where the microorganism produces new enzymatic machinery [38,39,40]. Based on the shape of experimental data, the following equation was proposed:

(14) $$E_{A}=\frac{1}{1+\exp\left(-r\left(t-t_{\mathrm{lag}}\right)\right)}$$

where t_lag_ (h) is the lag time experimentally observed. Figure 3b shows that E_A_ is an increasing function of time, tending to 1 when t ≫ t_lag_. In analogy with n, r is a shape factor that describes the gradual transition from the lag phase to the active phase of growth. A higher value of r implies a steeper change of E_A_ around t = t_lag_.

**Figure 4:**
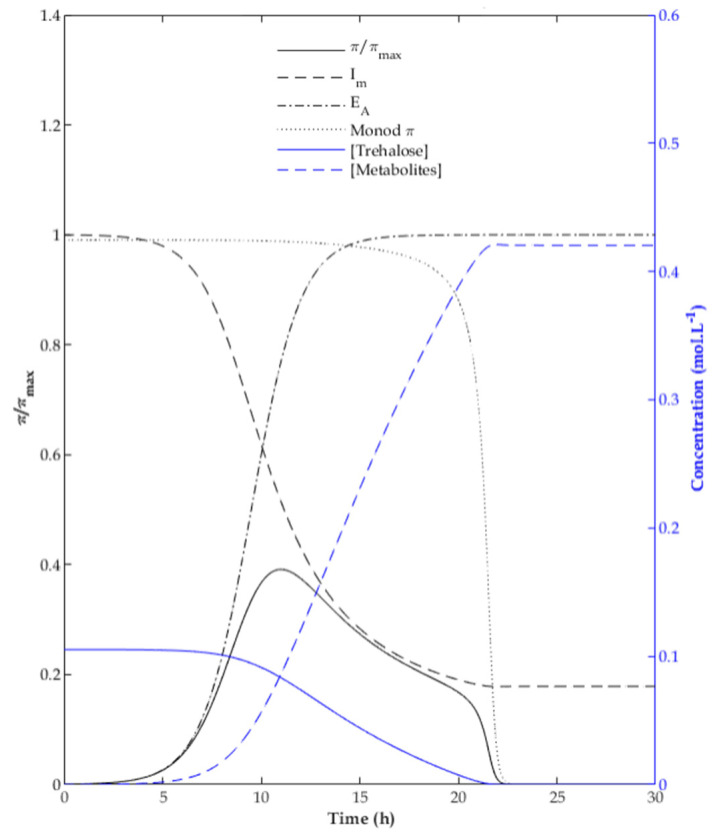
Typical evolution of the relative production rate over time.

To illustrate the features of the proposed model, a representation of the dimensionless specific growth and production rates (μ/μ_max_ and π/π_max_) over time is depicted in Figure 4. The dynamic behavior of both variables is similar given the similarity of Equations (1)–(5). The specific rates achieve a maximum value in the active growth phase, and they are zero when t ≪ t_lag_ and when the substrate is depleted. The shape of the curve is defined by three factors: in the increasing region (0 to 10 h in Figure 4), the dominant effect is enzyme activation E_A_ (Equation (14)); in the slowly decreasing region (10 to 20 h), the rate is controlled by inhibition (Equation (11) or (12)), whereas in the sharply decreasing region (20 to 22 h) it is controlled by substrate limitation, corresponding to the Monod-like factor in Equation (9) or (10).

### 3.2. Model Parameter Identification

The system of kinetic equations for a single fermentation experiment included 24 parameters: five yield coefficients, five inhibition parameters, five growth/production rates, five Monod-like saturation constants, three shape factors, and one lag time. Due to a limited number of experimental data and to facilitate the identification procedure, a single value was adopted for the inhibition parameter (K_Im_) and the Monod saturation constant (K_Sm_) of the four identified metabolites. Moreover, 10 parameters were fixed for all experiments: the shape factors, the yield coefficients, and the Monod saturation constants (K_SX_ and K_Sm_). For each fermentation, lag time was determined by graphical readout. This simplification of fixing parameters independent of operating conditions is supported by two assumptions often used in the literature: (1) metabolite production yields are constant and therefore independent of culture conditions [41] and (2) the saturation constant of the Monod model depends only on the nature of the substrate [33,38], which was the same in all experiments of this study.

The remaining group of seven parameters (μ_max,X_, π_max,F_, π_max,A_, π_max,L_, π_max,E_, K_IX_, K_Im_) were identified for each fermentation of the experimental design by nonlinear regression. Here, the Levenberg–Marquardt algorithm [42] was used to minimize the sum of squares of the errors between experimental and predicted concentrations. However, since the ranges and the number of measurements were slightly different among the metabolites, the values compared in the least squares function were normalized by dividing by their maximum value and were weighted by the relevant number of experimental measurements.

The quality of the model representation was quantified with two error indicators, defined as follows: 

Root mean square error:

(15) $$\mathrm{RMSE}={\left[\frac{1}{N}\overset{N}{\underset{i=1}{\sum }}{\left(C_{\mathrm{model},i\;}{-\;C}_{\exp,i}\right)}^{2}\right]}^{1/2}$$

Relative mean error (as a percentage):

(16) $$\mathrm{RME}=\frac{1}{N}\overset{N}{\underset{i=1}{\sum }}\frac{\left|C_{\mathrm{model},i}{\;-\;C}_{\exp,i}\right|}{C_{\exp,\max}{\;-\;C}_{\exp,\min}}\;\cdot 100\%$$

where N is the number of available measurements, C_model_ and C_exp_ are respectively the values of the concentration variables calculated with the model and measured experimentally.

### 3.3. Response Surface Model for Parameter Dependence on Fermentation Conditions

Nonlinear regression was performed to model the relationship between the seven parameters of the dynamic model specific to each experiment and the fermentation operating conditions—namely, temperature (T) and pH. The regression model had a similar form for all parameters, the logarithm of the parameter being expressed as a second-order polynomial with interaction:

(17) $$\log_{10}{\mathrm{Par}}_{i}=\beta_{0i}+\beta_{1i}T+\beta_{2i}\mathrm{pH}+\beta_{3i}T^{2}+\beta_{4i}{\mathrm{pH}}^{2}+\beta_{5i}\mathrm{TpH}$$

The regression coefficients (β) for all parameters depending on operating conditions (μ_max,X_, π_max,F_, π_max,A_, π_max,L_, π_max,E_, K_IX_, K_Im_) were simultaneously computed by least-squares optimization based on all available concentration measurements. In this way, the accuracy and standard errors of the coefficients were statistically acceptable, due to a large number of degrees of freedom: several hundreds of concentration data were used to estimate 42 coefficients. Initial guesses for these coefficients were obtained using Equation (17), and parameter values were determined separately for each experiment.

In this procedure, two sets of data from the experimental design were defined as indicated in Figure 2: eight calibration experiments, located in extreme positions of the experimental domain, used simultaneously for coefficients (β) estimation, and eight validation experiments, only used a posteriori to verify the accuracy of the complete dynamic model.

## 4. Results and Discussion

### 4.1. Model Parameter Identification

The values of the parameters that are independent of operating conditions, summarized in Table 1, were determined from the experimental data of experiment F10. This run was placed in a central position in the composite experimental design (T = 30 °C, pH = 8) (Figure 2). Monod saturation constants are usually difficult to determine from batch experiments because the number of measurements is typically very low in the substrate limitation zone. Saturation constants were thus fixed to a common value with a typical order of magnitude [43]. As for yields, they were found to differ from the theoretical ones defined through standard stoichiometric reactions of anabolism and catabolism. These differences can be due to other reactions involving the carbon substrate, whose products were not analytically measured and were not considered in the model.

After fixing the parameters in Table 1 for the whole set of experiments, the group of seven adjustable parameters of the model (μ_max,X_, π_max,F_, π_max,A_, π_max,L_, π_max,E_, K_IX_, K_Im_) were identified for each run by nonlinear regression.

The parameters obtained by this procedure are summarized in Table 2. Standard errors were computed from the variance–covariance matrix of the nonlinear optimization algorithm. These errors represented between 5% and 13% of the value of the identified parameters, a reasonable uncertainty level for a biological model.

For the whole set of experiments, the prediction errors are reported in Appendix A Table A1. Except for some runs for variables S, F, and A, all RME were lower than 15%. Additionally, the average RMSE and RME values for each concentration were of the same order of magnitude as the experimental variability, here defined as the biological repeatability for run F01, for which three independent replicates were performed. These results validate the formulation and accuracy of the proposed model under the operating conditions included in the experimental design.

**Figure 5:**
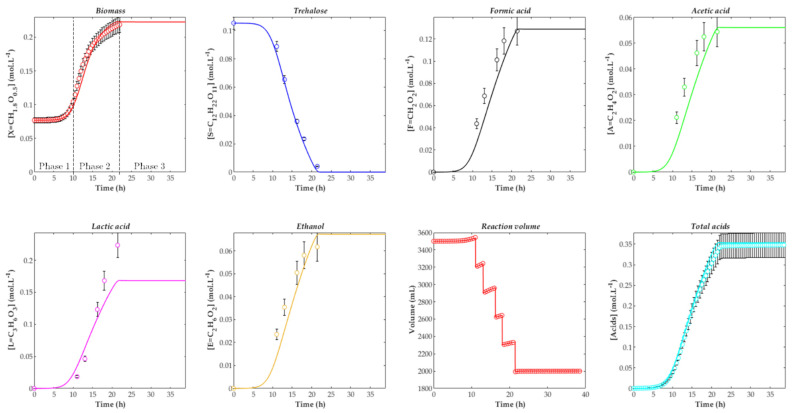
Evolution of concentrations over time for experiment F10 (T = 30 °C, pH = 8). Comparison between model (continuous line, using parameters from Table 1 and Table 2) and experimental data (symbols). The error bars for data represent the biological standard deviation, calculated from three independent repetitions of the run F01.

In the specific case of reference run F10, a comparison between the model simulation (using the corresponding parameters from Table 2) and experimental data is illustrated in Figure 5.

Three growth phases are apparent in Figure 5: a lag phase (phase 1, between 0 and 10 h); a phase of active growth, substrate consumption, and metabolite production (phase 2, between 10 h and 21 h); and a final phase where concentrations do not change over time, owing to the depletion of the carbon source or growth inhibition by metabolites (phase 3, after 21 h). Regarding culture volume evolution, as already mentioned, the discrete variations at regular intervals were due to sampling for analysis of the culture medium and the gradual increase was due to NaOH addition for pH control. One can also observe that the four metabolites were produced simultaneously, with no gap for the growth dynamics. The metabolites were thus primary end products generated during a single trophophase [44]. This justifies the choice of a global inhibitor concentration (Equation (13)), which included four correlated concentrations.

In consideration of the visual fit from Figure 5, the model representation is reasonably satisfactory. The most pronounced discrepancy between the model and experimental data appears for lactic acid, for which the model predicted a lower concentration before substrate depletion. This is related to a slightly underestimated yield factor Y_L/S_.

### 4.2. Response Surface Model for Parameter Dependence on Fermentation Conditions

Model parameters were expressed as a function of temperature and pH, according to the surface model (Equation (17)). The values of the β regression coefficients were adjusted globally using the whole set of calibration data.

**Figure 6:**
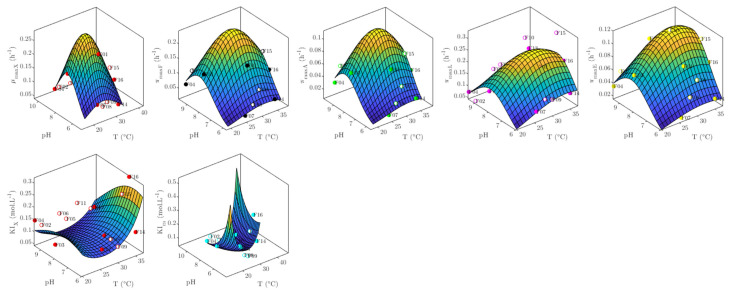
Response surfaces for model parameters, calculated with globally adjusted β coefficients in Equation (17).

The resulting response surfaces for the seven model parameters are plotted in Figure 6. For the five kinetic parameters, (i.e., the maximum specific growth and production rates), the response surfaces have the same convex shape, with a well-defined maximum value at intermediate T and pH conditions. These maxima likely indicate the optimal temperatures and pH for cellular growth, as well as the enzymatic activity catalyzing each of the reactions, leading to the production of the different metabolites (Figure 1).

Concerning the inhibition concentrations, the response surface for K_Im_ has a concave shape with a local minimum, whereas that of K_IX_ resembles a saddle surface. For this latter case, the surface shape indicates that for every pH there is a T where K_IX_ is minimal, and for every T there is a pH where K_IX_ is maximal. Both K_Im_ and K_IX_ represent the combined effect of several inhibiting metabolites (Equations (11)–(13)) with potentially different inhibition mechanisms.

**Figure A1:**
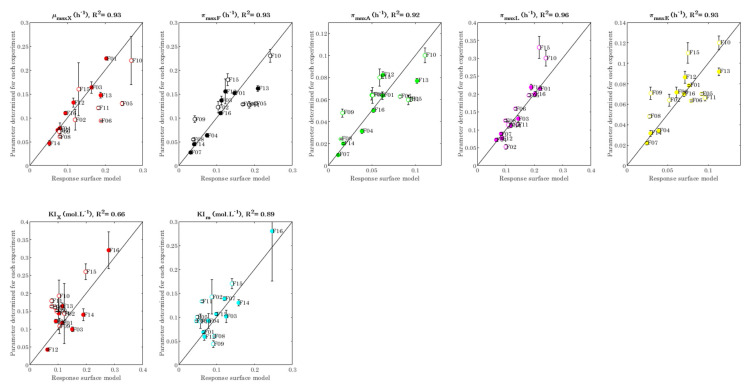
Comparison between model parameters determined for each experiment (Table 2) and parameters computed with the response surface models (Equation (17) using coefficients in Table A2).

For completeness, the final values of the regression coefficients of Equation (17) for the seven adjustable parameters of the dynamic model are reported in Appendix A Table A2. All coefficients in Equation (17) for each model parameter were significantly different from zero at a 0.05 level. A comparison between the parameter values determined for each experiment (Section 4.1) and the parameter values computed with Equation (17) (from globally adjusted β coefficients) is depicted in Appendix A Figure A1. The goodness of the fit was assessed through the coefficient of determination, *R*^2^. This coefficient is higher than 0.89 for six out of seven model parameters, which is a high threshold for biological data. In the case of K_Im_, only 66% of the variance of this parameter was explained by variables T and pH. The remaining 34% could be associated with inherent experimental variability and factors not included in the model, for instance transient variability of the inhibition and kinetics parameters and actual dependence of the fixed parameters (Table 1) with T and pH [45]. From a more general point of view, differences from experimental data could be due to features that were not represented by the mathematical model, such as population segregation, internal pH variability, and concentration gradients in the culture medium [46,47].

### 4.3. Model Validation 

The ability of the dynamic model including the parameters calculated from operating conditions (Equation (17)) to predict data of independent experiments was assessed with a set of validation experiments.

**Figure 7:**
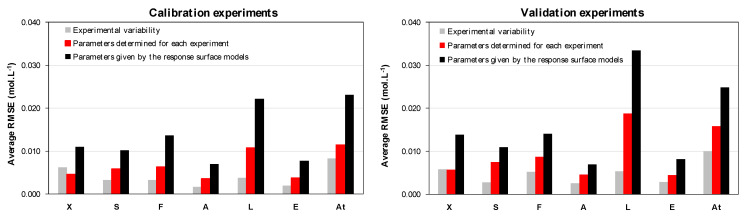
Comparison between experimental variability and average RMSE values for concentrations computed using parameters determined for each experiment (Table 2) and the response surface models (Table A2 and Equation (17)).

A comparison between the average RMSE values obtained in Section 4.1 (determined for each experiment) and Section 4.2 (calculated from operating conditions) for calibration and validation sets is depicted in Figure 7. In most cases, RMSE values were higher than the corresponding experimental variabilities, indicating that more complex models could capture additional phenomena not included in the present model, such as dependence of yields, saturation constants, or lag time (Table 1) on operating conditions. As one might expect, RMSE was generally lower for the calibration experiments than for the validation experiments, not used for parameter determination. However, the relative difference remained small (less than 30%), indicating a satisfactory ability of the developed model to predict time evolution of the considered biomass, substrate, and metabolites under new conditions within the explored experimental range.

It also appears in Figure 7 that average RMSE values with parameters given by the response surface model (Table A3) are about 50% higher than with parameters determined separately for each experiment (Table A1), for both calibration and validation sets. This result could be expected since in the global calibration step, data from eight independent experiments were combined as a whole for the least squares estimation, with a detrimental effect on the individual representation of each experiment. However, results with the parameters calculated from operating conditions are the most useful in engineering purposes since they enable a quick prediction of growth and metabolites production dynamics, based on the selected combination of temperature and pH.

In light of this quantitative analysis, the prediction accuracy of the empirical dynamic model coupled to the regression model may be considered satisfactory within the operating domain covered in this study.

### 4.4. Model-Based Optimization of Fermentation Operating Conditions for Industrial Use

Optimal conditions for growth and metabolite production of *C. maltaromaticum* calculated using the developed model are summarized in Table 3. Two optimization criteria were considered: final concentrations and final productivities calculated for a 99.9% substrate consumption.

**Figure A2:**
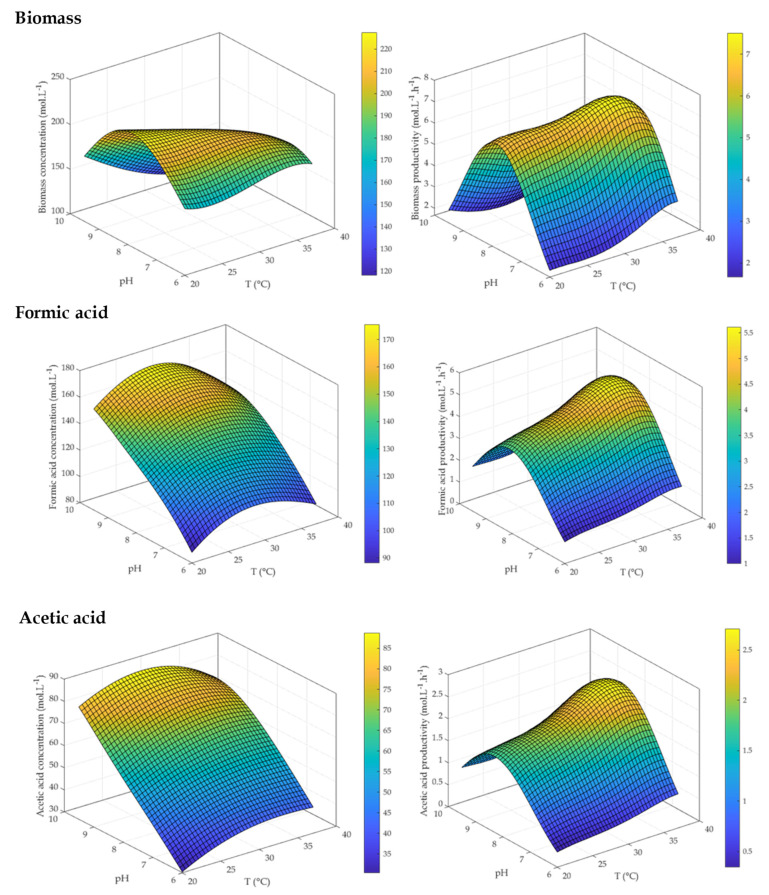
Evolution of final concentrations (left) and batch-average productivities (right) with temperature and pH for biomass, formic acid, and acetic acid.

**Figure A3:**
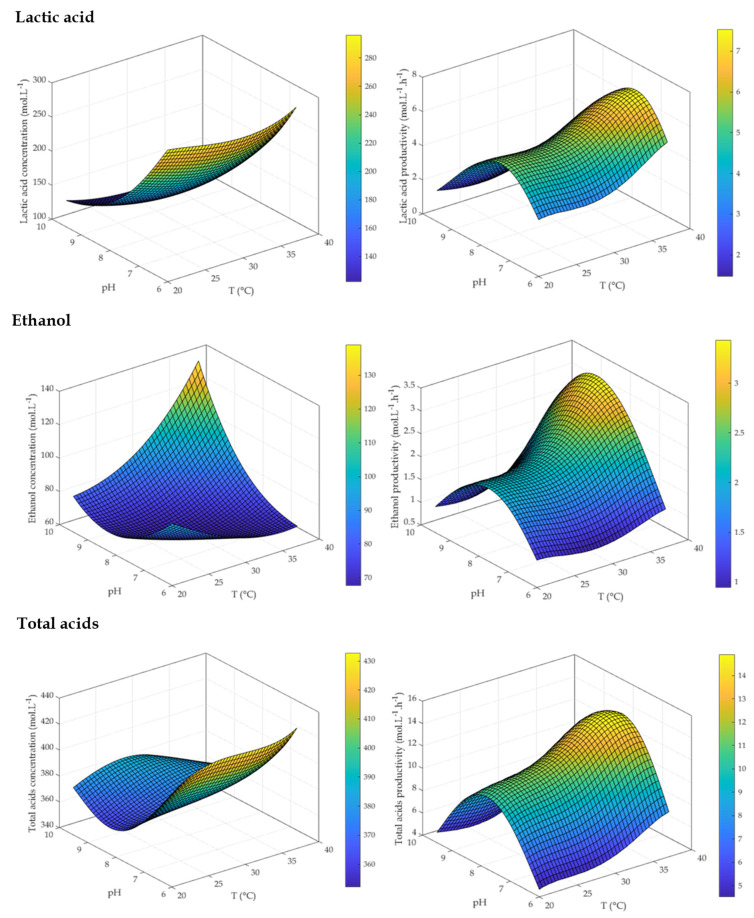
Evolution of final concentrations (left) and batch-average productivities (right) with temperature and pH for lactic acid, ethanol, and total acids.

For a detailed representation of the evolution of final concentrations and productivities for biomass and metabolites with temperature and pH, the reader is referred to Appendix A Figure A2 and Figure A3. As a general trend, the highest productivities were obtained around 35 °C and pH 7.5, although the exact optimal conditions depended on the considered metabolite (Table 3). No general trend was readily apparent for the maximization of the final concentrations.

These data can be useful in optimizing industrial processes involving the growth of *C. maltaromaticum* cells in a trehalose-based substrate. A first application consists of producing *C. maltaromaticum* concentrates, regardless of metabolite production. In this case two conditions of cultivation appear advisable: 20 °C and pH 7.8 to maximize concentration (227 mmol_C_·L^−1^) or 33.5 °C and pH 7.5 in order to maximize productivity (7.49 mmol_C_·L^−1^·h^−1^) and thus the biomass production per unit of time, at the expense of a 12% reduction of the final biomass concentration (199 mmol_C_·L^−1^).

A second application deals with the development and parametrization of time–temperature integrators (TTI), labels in which a pH decline, associated with acids synthesis, entails an irreversible color change from green to red. Modulating the acidifying activity of *C. maltaromaticum* thus allows a reliable shelf-life estimation of different food products. Long shelf-lives can be tracked using TTI composed of concentrates exhibiting low acidifying activities (minimal production of total acids), while short shelf-lives can be tracked using concentrates exhibiting high acidifying activities. In the scenario of maximizing acidifying activity, the production of total acids must be favored, and thus fermentation should be carried out under two possible conditions: 37.0 °C and pH 6.0 to maximize their final concentration (433 mmol·L^−1^) or 35.5 °C and pH 7.7 to maximize their productivity (14.90 mmol·L^−1^·h^−1^). Under these conditions, the biomass production decreases respectively by 20% and 4% with respect to its optimal values. If the objective is, on the contrary, to minimize acidifying activity, two conditions can be envisaged to favor the lowest production of total acids: 37.0 °C and pH 9.5 for a final concentration of 352 mmol L^−1^ or 25.0 °C and pH 9.5 for a final productivity of 4.53 mmol·L^−1^·h^−1^. Under these conditions, the mean biomass production would decrease respectively by 48% and 77% with respect to the maximal values.

Data from Table 3 show that the conditions to minimize the total acids concentration (37.0 °C and pH 9.5) coincide with those to maximize the ethanol concentration (the non-acidifying metabolite, 139 mmol L^−1^) and lead to a lactic acid concentration close to its minimal value (128 mmol L^−1^ versus the minimum around 120 mmol L^−1^). Conversely, when the production of total acids is maximized, the lactic acid concentration is also maximal (296 mmol L^−1^) and that of ethanol is close to its minimum (72 mmol L^−1^ versus 68 mmol L^−1^).

Furthermore, it should be noted that the condition 27 °C and pH 7.6 leads both to a good biomass productivity (6.93 mmol·L^−1^·h^−1^ versus the maximum 7.49 mmol·L^−1^·h^−1^) and a low total acids concentration (372 mmol L^−1^ versus the minimum 352 mmol L^−1^). Cultivation under this condition turns out be advantageous to ally a high biomass production and a relatively low total acidification.

## 5. Conclusions

The dynamic model developed in this study is able to predict with satisfactory accuracy the growth of *C. maltaromaticum* CNCM I-3298 (average error of 10%) as well as the conversion of trehalose into four primary metabolites (average error of 14%) under a wide range of conditions of temperature and pH. The interpolation capability of the model was verified with a set of eight independent validation experiments, for which the average relative error was 13%.

This model constitutes a useful tool for optimizing *C. maltaromaticum* cultures. Based on two easily controllable parameters, pH and temperature, it could be implemented in industrial applications of food technology to define optimal growth and metabolite production conditions with various objectives, such as the maximization of biomass for production of bacterium concentrates or the maximization or minimization of the acidifying activity. A typical operating condition for this bacterium could be, for instance, 30.0 °C and pH 7.0. If the goal is to produce bacterium concentrates, to maximize final biomass concentration, our results suggest that a quite different condition should be selected (20.0 °C and pH 7.8), while for maximum biomass productivity, 33.5 °C and pH 7.5 is most appropriate. Such results are quite difficult to anticipate from the qualitative knowledge of the bacterium alone, and a large number of time-consuming experiments would be required to locate these optimal conditions experimentally without constructing a dynamic model of the process.

The effort of developing the model is especially cost effective when a variety of scenarios are explored. If the goal is to develop time–temperature integrators (TTI) to track the cold-chain of food products, a set of labels with specific shelf-lives has to be designed for various target products. The range of desired shelf-lives can be as large as 1 to 30 days, requiring very different TTI designs. In a traditional approach, for each desired shelf-life duration, a range of factors such as the initial bacterium concentration and the buffer properties of the medium have to be explored in a series of relatively time-consuming experiments. In such an environment, temperature varies in an arbitrary but known way, and pH depends on the produced acids. The presented dynamic model can be extended to predict the moment when a specific amount of acids is produced, corresponding to the pH-induced color change of the TTI label and hence to the desired shelf-life. Model-based design of the TTI labels is expected to be faster and more accurate than a trial and error procedure.

On a more fundamental level, further work is required to incorporate the effect of other culture parameters, such as aeration, nutrient concentrations, or the use of a different carbon source, which may modify growth kinetics and metabolite production. Additionally, it would be relevant to deepen the understanding of inhibition mechanisms of the metabolites to give more biological significance to the associated parameters in the model.

## Figures and Tables

**Table 1:** Model parameters independent of operating conditions, determined from the experimental data of experiment F10 (T = 30 °C, pH = 8) with t_lag_ = 10 h.

Parameter	Constant Value
Y_X/S_ (mol_C_.mol^−1^)	6.9
Y_F/S_ (mol.mol^−1^)	5.6
Y_A/S_ (mol.mol^−1^)	3.8
Y_L/S_ (mol.mol^−1^)	7.0
Y_E/S_ (mol.mol^−1^)	4.7
K_SX_ (mol L^−1^)	0.001
K_Sm_ (mol L^−1^)	0.001
*n*	3
*p*	1
r (h^−1^)	0.8

**Table 2:** Model parameters determined for each experiment by nonlinear regression.

Fermentation		µ_maxX_ (h^−1^)	π_maxF_ (h^−1^)	π_maxA_ (h^−1^)	π_maxL_ (h^−1^)	π_maxE_ (h^−1^)	KI_X_ (Mol.L^−1^)	KI_m_ (Mol.L^−1^)
F01	Value	0.224	0.152	0.064	0.215	0.078	0.117	0.069
	Standard error	0.003	0.003	0.003	0.005	0.001	0.004	0.003
F02	Value	0.096	0.122	0.064	0.053	0.064	0.143	0.142
	Standard error	0.021	0.012	0.007	0.006	0.006	0.083	0.036
F03	Value	0.164	0.137	0.064	0.131	0.072	0.099	0.102
	Standard error	0.012	0.009	0.004	0.009	0.005	0.006	0.013
F04	Value	0.078	0.063	0.031	0.072	0.034	0.144	0.092
	Standard error	0.012	0.004	0.002	0.004	0.002	0.047	0.016
F05	Value	0.130	0.130	0.060	0.196	0.070	0.160	0.100
	Standard error	0.005	0.002	0.002	0.004	0.001	0.004	0.004
F06	Value	0.094	0.129	0.063	0.159	0.063	0.163	0.092
	Standard error	0.001	0.002	0.001	0.003	0.001	0.003	0.002
F07	Value	0.074	0.028	0.010	0.089	0.022	0.122	0.139
	Standard error	0.002	0.001	0.001	0.004	0.002	0.005	0.004
F08	Value	0.060	0.055	0.024	0.126	0.048	0.149	0.060
	Standard error	0.002	0.002	0.000	0.003	0.002	0.001	0.002
F09	Value	0.071	0.097	0.048	0.114	0.071	0.108	0.044
	Standard error	0.009	0.007	0.004	0.010	0.006	0.021	0.007
F10	Value	0.220	0.230	0.100	0.300	0.120	0.193	0.091
	Standard error	0.051	0.013	0.007	0.022	0.007	0.044	0.015
F11	Value	0.121	0.127	0.060	0.116	0.066	0.179	0.133
	Standard error	0.004	0.007	0.004	0.005	0.003	0.006	0.003
F12	Value	0.132	0.155	0.082	0.076	0.086	0.043	0.059
	Standard error	0.010	0.026	0.003	0.002	0.006	0.002	0.007
F13	Value	0.147	0.162	0.077	0.219	0.092	0.164	0.106
	Standard error	0.006	0.006	0.002	0.008	0.004	0.009	0.003
F14	Value	0.047	0.045	0.020	0.112	0.032	0.140	0.130
	Standard error	0.005	0.004	0.001	0.006	0.003	0.016	0.007
F15	Value	0.160	0.180	0.080	0.330	0.110	0.260	0.170
	Standard error	0.056	0.013	0.008	0.030	0.010	0.022	0.010
F16	Value	0.110	0.110	0.050	0.200	0.070	0.320	0.280
	Standard error	0.003	0.004	0.002	0.007	0.003	0.051	0.104

**Table 3:** Optimal conditions for growth and production of metabolites according to the developed model. **In bold**: targeted metabolite for each set of operating conditions. Final concentrations and productivities calculated with initial conditions: [X]_0_ = 80 mmol L^−1^, [S]_0_ = 100 mmol L^−1^, [F, A, L, E]_0_ = 0.

A. Target		T (°C)	pH	Final Concentrations (mmol L^−1^)						Final Productivities (mmol L^−1^.h^−1^)					
				X	F	A	L	E	A_T_	X	F	A	L	E	A_T_
Biomass	B_conc._↑	20.0	7.8	**227**	129	56	177	73	363	6.56	3.73	1.61	5.11	2.09	10.46
Formic acid	F_conc._↑	28.0	9.5	133	**176**	89	123	94	387	1.68	2.20	1.12	1.54	1.18	4.86
Acetic acid	A_conc._↑	28.0	9.5	133	176	**89**	123	94	387	1.68	2.20	1.12	1.54	1.18	4.86
Lactic acid	L_conc._↑	37.0	6.0	180	95	42	**296**	72	433	1.68	2.20	1.12	1.54	1.18	4.86
Ethanol	E_conc._↑	37.0	9.5	118	147	77	128	**139**	352	2.35	2.90	1.54	2.54	2.76	6.98
Ethanol	E_conc._↓	27.0	7.6	217	143	62	166	**68**	372	6.93	4.57	1.98	5.31	2.16	11.86
Total acids	A_Tconc._↑	37.0	6.0	180	95	42	296	72	**433**	3.33	1.76	0.77	5.48	1.33	8.01
Total acids	A_Tconc_.↓	37.0	9.5	118	147	77	128	139	**352**	2.35	2.90	1.54	2.54	2.76	6.98
Biomass	B_prod._↑	33.5	7.5	199	139	64	178	73	382	**7.49**	5.25	2.41	6.71	2.77	14.38
Formic acid	F_prod._↑	34.5	8.0	178	148	71	163	82	381	6.77	**5.61**	2.70	6.18	3.12	14.48
Acetic acid	A_prod._↑	35.0	8.1	172	148	72	161	85	381	6.49	5.58	**2.71**	6.06	3.21	14.35
Lactic acid	L_prod._↑	37.0	7.1	188	123	58	215	78	395	6.60	4.31	2.04	**7.54**	2.73	13.89
Ethanol	E_prod._↑	37.0	8.3	158	144	73	162	97	378	5.57	5.08	2.56	5.70	**3.43**	13.34
Ethanol	E_prod._↓	28.0	6.0	175	108	41	278	78	427	2.14	1.32	0.50	3.38	**0.95**	5.20
Total acids	A_Tprod._↑	35.5	7.7	184	139	67	177	81	384	7.16	5.41	2.59	6.90	3.13	**14.90**
Total acids	A_Tprod._↓	25.0	9.5	146	173	88	126	87	386	1.71	2.03	1.03	1.48	1.02	**4.53**

↑ maximization, ↓ minimization, conc.: final concentration, prod.: batch-average productivity.

## Data Availability

The data presented in this study are available on request from the corresponding author. The data are not publicly available due to an ongoing research project.

## Nomenclature

A	Acetic acid
E	Ethanol
F	Formic acid
L	Lactic acid
S	Carbon substrate (trehalose)
X	Biomass
E_A_	Enzymatic activation factor
C (mol·L^−1^)	Concentration (in the calculation of errors and the definition of the inhibition factors)
[i] (mol·L^−1^)	Concentration of species i (substrate, metabolite, biomass) in the culture medium (in the system of differential equations)
I_m_	Production inhibition factor of metabolite m
I_X_	Biomass growth inhibition factor
K_Im_ (mol·L^−1^)	Concentration for 50% production rate inhibition of metabolite m
K_IX_ (mol_C_·L^−1^)	Concentration for 50% growth rate inhibition of biomass
K_Sm_ (mol·L^−1^)	Concentration of production rate saturation of metabolite m
K_SX_ (mol_C_·L^−1^)	Concentration of biomass growth rate saturation
mol_C_	Carbon-mol of biomass
n	Shape factor of the growth inhibition function
p	Shape factor of the production inhibition function
pH	Potential of hydrogen
Q (L·h^−1^)	Rate of base addition for pH control
R	Shape factor of the enzymatic activation function
T (K)	Temperature
TTI	Time–temperature indicator
RMSE	Root-mean square error
RME	Relative mean error
SE	Standard error
t (h)	Time
t_lag_ (h)	Lag time
V (L)	Culture medium volume
Y_i/S_ (mol·mol^−1^)	Yield of product i on substrate S
Y_X/S_ (mol_C_·mol^−1^)	Biomass yield on substrate S
μ_X_ (h^−1^)	Specific growth rate
μ_max,X_ (h^−1^)	Maximal specific growth rate
π_m_ (h^−1^)	Specific production rate of metabolite m
π_max,m_ (h^−1^)	Maximum specific production rate of metabolite m

## Appendix A

### Appendix A.1. Model Parameter Identification

**Table A1:** Residual modelling error with model parameters determined for each experiment and summarized in Table 2.

	RMSE (Mol L^−1^)							RME (%)						
	X	S	F	A	L	E	A Total	X	S	F	A	L	E	A Total
**F01**	0.008	0.006	0.010	0.007	0.022	0.007	0.033	4	4	7	13	6	10	7
**F02**	0.005	0.007	0.006	0.003	0.004	0.003	0.005	4	16	8	8	8	7	3
**F03**	0.005	0.006	0.003	0.001	0.003	0.001	0.006	4	9	3	2	3	2	1
**F04**	0.006	0.006	0.003	0.001	0.005	0.002	0.005	7	13	5	5	6	6	3
**F05**	0.008	0.005	0.014	0.007	0.027	0.007	0.034	4	4	9	11	10	9	8
**F06**	0.006	0.004	0.012	0.005	0.024	0.006	0.012	5	2	7	7	8	8	3
**F07**	0.006	0.007	0.011	0.006	0.009	0.006	0.007	4	5	14	33	4	11	2
**F08**	0.006	0.007	0.011	0.005	0.030	0.004	0.003	4	5	17	23	8	7	1
**F09**	0.004	0.005	0.002	0.001	0.002	0.002	0.003	7	17	6	7	5	6	2
**F10**	0.005	0.009	0.006	0.004	0.028	0.004	0.026	3	7	4	7	9	5	6
**F11**	0.003	0.018	0.009	0.005	0.011	0.006	0.032	3	27	9	10	9	11	6
**F12**	0.005	0.013	0.002	0.001	0.003	0.001	0.004	13	61	3	4	8	2	2
**F13**	0.002	0.006	0.012	0.005	0.024	0.005	0.007	2	4	7	6	6	5	2
**F14**	0.003	0.003	0.009	0.005	0.012	0.006	0.015	4	3	16	20	5	12	4
**F15**	0.009	0.005	0.009	0.006	0.023	0.005	0.011	5	4	7	13	8	8	2
**F16**	0.003	0.001	0.001	0.001	0.008	0.001	0.014	2	1	2	4	6	2	3
**Mean**	0.005	0.007	0.008	0.004	0.015	0.004	0.014	5	11	8	11	7	7	4

Response surface model for parameter dependence on fermentation conditions.

**Table A2:** Response surface coefficients fitted to experimental data by multiple regression.

Variable	Coefficient	µ_maxX_ (h^−1^)	π_maxF_ (h^−1^)	π_maxA_ (h^−1^)	π_maxL_ (h^−1^)	π_maxE_ (h^−1^)	KI_X_ (Mol L^−1^)	KI_m_ (Mol L^−1^)
Constant	Value (*β*_0_)	−1.38 × 10^1^	−1.64 × 10^1^	−1.74 × 10^1^	−1.21 × 10^1^	−1.12 × 10^1^	−8.61 × 10^−1^	1.19 × 10^1^
	Standard error	0.05 × 10^1^	0.07 × 10^1^	0.09 × 10^1^	0.09 × 10^1^	0.07 × 10^1^	0.05 × 10^−1^	0.03 × 10^1^
T	Value (*β*_1_)	2.63 × 10^−1^	2.66 × 10^−1^	2.71 × 10^−1^	1.82 × 10^−1^	1.27 × 10^−1^	−1.87 × 10^−1^	−5.80 × 10^−1^
	Standard error	0.13 × 10^−1^	0.26 × 10^−1^	0.28 × 10^−1^	0.23 × 10^−1^	0.14 × 10^−1^	0.05 × 10^−1^	0.17 × 10^−1^
pH	Value (*β*_2_)	2.42 × 10^0^	2.89 × 10^0^	3.01 × 10^0^	2.27 × 10^0^	2.02 × 10^0^	6.65 × 10^−1^	−1.27 × 10^0^
	Standard error	0.11 × 10^0^	0.14 × 10^0^	0.19 × 10^0^	0.16 × 10^0^	0.13 × 10^0^	0.45 × 10^−1^	0.16 × 10^0^
T²	Value (*β*_3_)	−4.26 × 10^−3^	−3.45 × 10^−3^	−3.26 × 10^−3^	−2.05 × 10^−3^	−1.92 × 10^−3^	3.97 × 10^−3^	7.95 × 10^−3^
	Standard error	0.13 × 10^−3^	0.32 × 10^−3^	0.33 × 10^−3^	0.81 × 10^−3^	0.22 × 10^−3^	0.09 × 10^−3^	0.32 × 10^−3^
pH²	Value (*β*_4_)	−1.46 × 10^−1^	−1.63 × 10^−1^	−1.66 × 10^−1^	−1.34 × 10^−1^	−1.22 × 10^−1^	−4.03 × 10^−2^	4.35 × 10^−2^
	Standard error	0.06 × 10^−1^	0.10 × 10^−1^	0.12 × 10^−1^	0.10 × 10^−1^	0.09 × 10^−1^	0.31 × 10^−2^	0.55 × 10^−2^
T·pH	Value (*β*_5_)	−3.53 × 10^−3^	−7.58 × 10^−3^	−9.17 × 10^−3^	−7.22 × 10^−3^	−4.68 × 10^−3^	−3.54 × 10^−3^	1.86 × 10^−2^
	Standard error	0.11 × 10^−3^	0.97 × 10^−3^	1.17 × 10^−3^	0.91 × 10^−3^	0.05 × 10^−3^	0.06 × 10^−3^	0.01 × 10^−2^

### Appendix A.2. Model Validation

Error indicators RMSE and RME for calibration and validation sets are summarized in Appendix A Table A3. The RME values varied from 1% to 53%, with an average of 15% for calibration set and 13% for validation set. Likewise, the average RME was lower than 15% for most of experiments, except for runs F12 (17%), F15 (18%), F09 (20%), and F16 (34%). Considering both calibration and validation sets, the average RME was 10% for biomass and 14% for substrate and metabolites.

**Table A3:** Quality of fit of the model with parameters computed with the response surface models.

Fermentation		RMSE (Mol L^−1^)							RME (%)						
		X	S	F	A	L	E	At	X	S	F	A	L	E	At
**Calibration**	**F01**	0.010	0.010	0.021	0.010	0.041	0.014	0.033	5	6	14	20	10	18	7
	**F03**	0.021	0.009	0.008	0.005	0.008	0.003	0.012	15	14	7	9	7	5	4
	**F04**	0.005	0.010	0.009	0.006	0.007	0.005	0.015	5	26	18	23	9	17	9
	**F07**	0.012	0.009	0.010	0.006	0.015	0.006	0.009	9	8	13	31	5	10	3
	**F12**	0.003	0.011	0.008	0.005	0.003	0.004	0.009	8	53	13	16	8	11	6
	**F13**	0.010	0.007	0.006	0.003	0.036	0.003	0.011	7	5	4	4	8	4	2
	**F14**	0.015	0.002	0.013	0.007	0.004	0.009	0.012	20	2	23	28	2	16	3
	**F16**	0.011	0.023	0.033	0.014	0.064	0.018	0.085	9	33	50	45	48	38	18
	**Mean**	0.011	0.010	0.014	0.007	0.022	0.008	0.023	10	18	18	22	12	15	7
**Validation**	**F02**	0.005	0.003	0.017	0.009	0.008	0.009	0.007	5	8	20	22	15	21	4
	**F05**	0.011	0.008	0.007	0.006	0.045	0.005	0.016	7	7	4	8	13	8	4
	**F06**	0.008	0.009	0.013	0.005	0.044	0.007	0.032	6	5	7	6	11	9	6
	**F08**	0.009	0.010	0.012	0.006	0.026	0.008	0.003	7	8	19	25	8	14	1
	**F09**	0.011	0.006	0.005	0.004	0.018	0.004	0.009	19	22	15	24	35	15	7
	**F10**	0.021	0.014	0.025	0.011	0.063	0.014	0.044	11	10	17	18	20	20	10
	**F11**	0.018	0.013	0.007	0.003	0.017	0.004	0.050	16	20	7	6	12	8	9
	**F15**	0.028	0.023	0.026	0.010	0.047	0.014	0.036	15	20	22	21	17	21	7
	**Mean**	0.014	0.011	0.014	0.007	0.033	0.008	0.025	11	13	14	16	16	15	6

Model-based optimization of fermentation conditions.
